# Development of a questionnaire to evaluate the management of gestational diabetes mellitus patients among obstetric nurses

**DOI:** 10.3389/fpubh.2025.1521673

**Published:** 2025-04-23

**Authors:** Yongyan Zhu, Yumin Ren, Jing Ma, Feifei Wan, Feifan Liu, Fangfang Du, Weili Li, Tian Tian, Xiaoqian Liu, Lili Li, Zhumin Jia

**Affiliations:** ^1^Department of Nursing, Henan Vocational College of Tuina, Luoyang, China; ^2^College of Nursing, Henan University of Science and Technology, Luoyang, China; ^3^Third Affiliated Hospital of Henan University of Science and Technology, Luoyang, China; ^4^First Affiliated Hospital of Henan University of Science and Technology, Luoyang, China

**Keywords:** obstetric nurses, gestational diabetes mellitus, management competence, questionnaire development, validity and reliability

## Abstract

**Background:**

The role of obstetric nurses in the management of gestational diabetes mellitus (GDM) is becoming increasingly important, and their management level will directly affect the patient’s health education and even the pregnancy outcome. However, there is a lack of tools to measure the competencies of GDM management among obstetric nurses.

**Methods:**

The questionnaire was developed in two stages: (a) creation of an initial questionnaire based on the definition of competency, literature review, and semi-structured interviews, followed by two rounds of Delphi expert inquiry; (b) evaluation of the questionnaire via piloting study on 30 obstetric nurses and testing of validity and reliability on 239 obstetric nurses.

**Results:**

A questionnaire containing dimensions of professional knowledge, professional skills, professional competence and personal attitude was developed. Correlation analysis revealed statistically significant correlations (*p* < 0.01) between each item and the total score, although two items had *r*-values <0.4, indicating weak correlations, so they were removed. The initial Cronbach’s *α* coefficient of 0.970 indicated that no items needed to be excluded. Five experts evaluated the content validity, yielding an S-CVI/Ave of 0.95 and the I-CVI values range from 0.80 to 1.00, meeting the reference standard. The Cronbach’s *α* coefficients for the four dimensions ranged from 0.793 to 0.928, while the overall Cronbach’s *α* coefficient of the questionnaire was 0.970. The retest reliability was 0.907, and the fold-half reliability was 0.950. The results of the reliability tests all met the measurement requirements of the questionnaire and could be used to evaluate the competency of obstetric nurses in the management of GDM patients.

**Conclusion:**

This study developed an assessment tool for evaluating the management ability of obstetric nurses for GDM patients, which consists of four dimensions and 35 items. The dimensions and items demonstrate the content of the competence theory, and the questionnaire shows good stability and reliability and validity.

**Recommendations for practice:**

It is recommended that healthcare institutions integrate this questionnaire into training and evaluation programs for obstetric nurses to improve care quality for GDM patients. Regular use will ensure nurses have the knowledge, skills, and attitudes needed to provide optimal care.

## 1 Introduction

Gestational diabetes mellitus (GDM) is one of the most common medical conditions affecting pregnant women (1, 2), the prevalence of which has risen dramatically worldwide with the rapid socioeconomic and lifestyle transformation (3, 4). According to data from International Diabetes Federation (IDF) and World Health Organization (WHO), the global incidence of GDM in recent years is 16.5%–17.0% (5), and that of China is 17.5%–18.9% (6, 7). Scientific and standardized management is crucial for maintaining normal blood glucose levels and improving the prognosis of GDM patients (8).

With regard to gestational diabetes mellitus management (GDMM), an abundance of guidelines have been formulated by the International Diabetes Federation, the World Health Organization, the United States (9), the United Kingdom, Canada and New Zealand (10, 11). At present, the GDMM in developed countries is relatively mature, management centers or multidisciplinary clinics are set up in hospitals (12, 13). For example, the National Health Service (NHS) system administers all public hospitals and unifies GDM outpatient management in the UK (14). The development of GDMM in China is relatively late, and the management mode is diversified (14), it is difficult to form a unified standard and mode. At present, the GDMM in China is mainly concentrated in the outpatient department, and the managers are mainly obstetricians (15), but there are some problems such as limited communication time and poor management effect. With the increasing number of GDM patients and the increasing demand for GDMM, obstetric nurses have gradually participated in the GDMM in recent years, and even played a leading role in it (16). In addition, the delivery management and postpartum management of GDM patients in most domestic hospitals are mainly completed by obstetric medical staff. Whether patients can get timely and standardized management is closely related to the GDMM level of obstetric medical staff.

Previous studies have shown that although obstetrical medical staff have a certain degree of GDM knowledge, their knowledge is not comprehensive, especially since the scores of obstetrical nurses on GDM-related knowledge are significantly lower than that of doctors. Obstetric nurses play a critical role as consultants, educators, and coordinators in the GDMM (17). As consultants, they provide essential guidance to pregnant women with GDM and their caregivers on key aspects, including diet, exercise, and blood glucose monitoring. As educators, they deliver comprehensive instruction on blood glucose management, dietary recommendations during pregnancy, postpartum lifestyle modifications, and psychological care for GDM patients. As coordinators, obstetric nurses facilitate effective communication and collaboration among GDM patients, their caregivers, obstetricians, nutritionists, and other healthcare professionals involved in the care process. A survey on GDM nursing knowledge, attitude and behavior of obstetric nurses in a hospital in Shandong Province was conducted, and the results showed that the score of GDM knowledge for obstetric nurses was only 50%, in particular, knowledge about GDM risk factors, disease complications, and effects of diseases on newborns were lack. Choi et al. (18) investigated the knowledge of nurses about GDM by using a 30-item knowledge questionnaire about GDM consisting of eight areas, and the results showed that the mean score of knowledge on GDM was 23.18. The results of this study suggest that nurses should be trained both systematically and individually on GDM to enhance their ability to detect and prevent the condition. This training would also increase their confidence in educating patients about GDM.

The GDMM level of obstetrical nurses directly affects the health education effect of GDM patients and even affects the pregnancy outcome (19, 20). However, at present, except for a few scholars’ investigation and research on the knowledge level of obstetric nurses, no relevant research has been found on the evaluation of their comprehensive management level. To date, no tool has emerged to evaluate the GDMM among obstetric nurses. The lack of this tool may be a key barrier to improving the GDMM level of obstetric nurses. The objective of this study was to develop and validate a questionnaire to measure the GDMM among obstetric nurses based on the competency framework.

## 2 Methods

**Figure 1:**
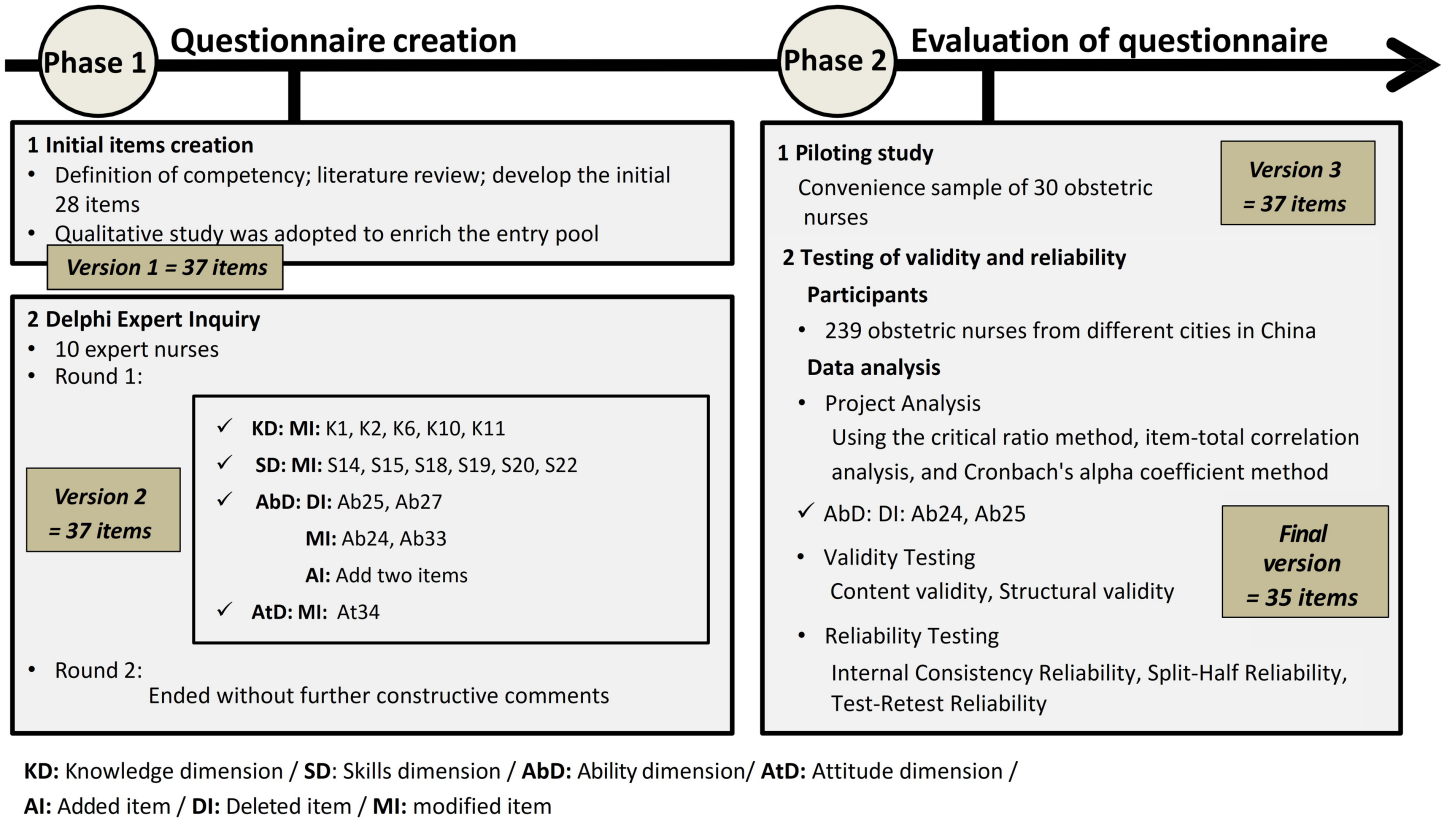
Global process of the questionnaire development.

We developed the questionnaire in two phases: Phase One was for the questionnaire creation, including initial items creation and Delphi expert inquiry; Phase Two was for the evaluation of questionnaire through piloting study and testing of validity and reliability. The global process of the questionnaire development is depicted in Figure 1.

Participants were given written information about the aim and the procedures of the study, and the right to withdraw at any time. Participants were assured that their names would not be used, and confidentiality would be maintained by the researchers. Before data collection, informed consent was obtained from each participant. Participation was voluntary.

### 2.1 Phase 1: questionnaire creation

This phase included two stages: (1) initial items creation and (2) Delphi expert inquiry.

#### 2.1.1 Initial items creation

The design of the questionnaire was based on the definition of competency as “the capacity of nurses to integrate cognitive, affective, and psychomotor abilities in nursing care provision” (21, 22). It encompasses a wide range of observable knowledge, skills, attitudes, and behavior patterns, which together constitute the capacity to provide a specific professional service (23). This study is grounded in the principles and standards of competency theory. Drawing upon the key elements of competency, the research team engaged in an extensive discussion, incorporating feedback from clinical nursing experts and reviewing relevant literature, to develop the corresponding competency assessment dimensions. We therefore consider that the GDMM requires obstetric nurses to advance their abilities in all domains of GDMM competence (i.e., knowledge, skills, attitudes/beliefs).

Firstly, a literature review was performed by taking “gestational diabetes,” “obstetrics and gynecology,” “obstetrics,” “nurses,” “nursing,” “management,” “questionnaire,” “scale” and “preparation/development” as subject terms in the databases of PubMed, Elsevier, EBSCO, Web of Science, Springer, CNKI, etc. The relevant literature was summarized, and a partial entries pool of the questionnaire was formed. In the initial stage, 28 items were developed with key elements of GDMM competence.

A qualitative study using in-depth, face-to-face interviews was adopted to enrich the entry pool, the purposive sampling was adopted. Eleven nurses and seven nurse managers at six hospitals in Luoyang City, China, completed the semi-structured interview between November 2021 and December 2021. Outlined interviews for obstetric nurses and obstetric nursing managers are listed in Supplementary Table S1. The interview recording and notes were sorted out in time after each interview; these interview data were summarized and analyzed using Colaizzi’s 7-step analysis method concerning phenomenological data (24). A total of 37 initial items (Version 1) were developed through literature review and qualitative interview.

#### 2.1.2 Delphi expert inquiry

An expert group, including 10 experts from Shanghai, Zhengzhou, Shenzhen, Luoyang, Xinxiang, and Handan, China, were selected to conduct two rounds of Delphi expert inquiry for the questionnaire. The experts must meet at least one of the criteria (a, b, c) and simultaneously meet criteria (d and e): (a) nursing education experts: engaged in nursing education for ≥5 years, with a postgraduate degree or an associate senior professional title or above; (b) obstetrics and gynecology experts: engaged in obstetric and gynecological clinical practice or nursing for ≥5 years, with a postgraduate degree or an associate senior professional title or above; (c) diabetes experts: engaged in diabetes clinical practice or nursing for ≥5 years, with a postgraduate degree or an associate senior professional title or above; (d) familiar with the current status of gestational diabetes management; (e) informed consent and voluntary participation in this study. These experts analyzed the dimensionality of the initial questionnaire (Version 1) and the adequacy of its items using the Delphi consensus technique (25–27). The group comprised 10 professionals with expertise in the fields of GDM education, care, management and clinical medicine. Each expert received the questionnaire by email along with a description of its objectives and dimensions. The experts assessed the adequacy of items, their relevance in the assigned dimension, and their comprehensibility, responding on a 5-point Likert scale. They were also asked to propose improvements in the wording of items or other aspects when appropriate.

The degree of consensus among experts is usually measured using the coefficient of variation (CV) and Kendall’s coefficient of concordance *W*. CV indicates the consistency of experts’ opinions on a particular item; the smaller the value, the better the coordination of expert opinions, with CV <0.25 being the standard. *W* measures the level of agreement among the rating data, indicating whether there is significant disagreement or divergence in experts’ opinions and evaluations of the questionnaire items. The value ranges from 0–1, with higher values indicating better coordination. Additionally, the *W* value needs to be subjected to a chi-square test; the smaller the *p*-value, the higher the reliability of the test for expert coordination (28).

The degree of consensus among experts is measured using the mean relevance score (*Mj*) and the full score rate (*Kj*) for each item. Using the Likert 5-point scale, the mean score should be above 3.5 points. The full score rate (*Kj*) can serve as a supplementary indicator; the higher the *Kj* value, the more experts rated the item as a full score, indicating the greater importance of the item. If an item does not meet both the mean score and the full score rate criteria, it should be deleted.

### 2.2 Phase 2: evaluation of the questionnaire

This phase included two stages: (1) pilot study and (2) testing of validity and reliability.

#### 2.2.1 Pilot study

A pilot study was conducted on 30 obstetric nurses [the number of participants in the pilot study should be three to five times the number of dimensions in the questionnaire (29), and the optimal sample size for testing typically ranges from 25 to 75 participants (30)] with professional experience of ≥1 years working in hospital engaged in obstetric care. The aim was to test whether the items of the questionnaire were suitable for obstetric nurses.

Comments of the obstetric nurses revealed no lack of clarity in the wording of the items. The items were readable, explicit, and accurate in reflecting the GDMM among obstetric nurses.

#### 2.2.2 Testing of validity and reliability

The number of nurses employed to validate this questionnaire was calculated based on an item-to-participant ratio of 1:5 to 1:10 (31). We included more nurses in consideration of missing data. Because the purpose of this survey was to develop a generalized instrument that could be used across a wide range of programs and organizations among nursing professionals, the sample was deliberately chosen to be as heterogeneous as possible, obstetric nurses who met the inclusion criteria were selected from Zhengzhou, Shenyang, Shenzhen, Dalian, Luoyang, Ordos, and other cities as research subjects. The recommended sample size for validation studies typically ranges from 200 to 400 participants (32). The survey was conducted between May 2022 and July 2022 in seven hospitals in China. Data were collected by the researchers using questionnaires at nurse meetings in hospital units and by email. The response rate across the hospitals was 96.37%; a total of 239 questionnaires were returned and analyzed.

After collecting the questionnaires, the data were verified by two individuals and manually entered into an Excel spreadsheet. Subsequently, the data were imported into SPSS 21.0 for analysis.

a) Project analysis

In this study, the items were analyzed using the critical ratio, homogeneity test, and Cronbach’s *α* coefficient, the corresponding methods were the critical ratio method, item-total correlation analysis, and Cronbach’s *α* coefficient method, respectively.

The critical ratio method, also known as the extreme group method, refers to the degree to which test items differentiate the content being studied. The higher the value, the stronger the discriminative ability of the item. Firstly, the total scores of the questionnaires are calculated and ranked in descending order. The top 27% are defined as the high-score group, and the bottom 27% as the low-score group. Independent samples *t*-tests are then used to analyze the items of both groups. When the decision value (CR value) shows a statistically significant difference (*p* < 0.05) and CR ≥3.0, it indicates that the item has good discriminative ability and should be retained; otherwise, it should be deleted.

The homogeneity test analysis uses the item-total correlation analysis method, which screens indicators based on the correlation coefficient between each item and the total score of the questionnaire. When the correlation coefficient *r* ≥ 0.4 and is statistically significant (*p* < 0.05), it indicates that the item has high homogeneity with the questionnaire and should be retained; otherwise, it should be deleted.

This study calculates the Cronbach’s *α* coefficient for each dimension and the questionnaire as a whole. If deleting a particular item increases the Cronbach’s *α* coefficient of the questionnaire, it indicates that the presence of that item reduces the internal consistency of the questionnaire. Thus, the item can be considered for deletion to improve the internal consistency of the questionnaire.

b) Validity testing

Content validity is primarily evaluated by experts based on the relevance and distribution of the questionnaire items. The content validity index (CVI) is used as a quantitative indicator, which includes the item-level CVI (I-CVI) for each item and the scale-level CVI/Ave (S-CVI/Ave), which is the average of all I-CVIs on the scale. The criteria for acceptance are I-CVI ≥0.78 and S-CVI/Ave ≥0.90. In this study, five experts were invited to rate the questionnaire. Each item was rated using a 4-point scale: 1 indicates “not relevant,” 2 indicates “slightly relevant,” 3 indicates “moderately relevant,” and 4 indicates “highly relevant.” The calculation formulas are as follows:


I−CVI=Number of experts rating3or4/Total number of experts

S−CVI/Ave=SumofallI−CVIs/Total number of items

Exploratory factor analysis (EFA) and confirmatory factor analysis (CFA) were used to examine the construct validity of the questionnaire. Pearson correlation coefficient was used to examine the relationship between each dimension and the overall questionnaire. A significance level of *p* < 0.05 was considered statistically significant. A correlation coefficient between 0.8 and 1.0 indicates a very strong correlation, between 0.6 and 0.8 indicates a strong correlation, and between 0.4 and 0.6 indicates a moderate correlation.

c) Reliability testing

The internal consistency of the questionnaire was assessed using Cronbach’s *α* coefficient. A Cronbach’s *α* ≥0.7 indicates good reliability, with higher values reflecting greater reliability (33).

The entire set of questionnaire items was randomly divided into two equal parts to test the correlation between the two halves. The Spearman–Brown coefficient was used for this assessment.

Test-retest reliability measures the stability and consistency of the questionnaire over time. After an initial completion of the questionnaire, a sample of 30 obstetric nurses was invited to complete the same questionnaire again after a 2-week interval. The correlation coefficients between the total scores and dimensions of the two administrations were calculated. Test-retest reliability should be above 0.7, with higher coefficients indicating greater consistency and stability of the questionnaire.

The acceptability of the questionnaire was evaluated based on factors such as the effective response rate, effectiveness, and the time required to complete the questionnaire.

### 2.3 Ethics approval

All the participants gave written informed consent to participate in the study and were assured of the anonymity and confidentiality of their information. Moreover, this study was approved by the Medical Ethics Committee of the First Affiliated Hospital of Henan University of Science and Technology (Ethical code: 2023-372).

## 3 Results

### 3.1 Phase 1: questionnaire creation

#### 3.1.1 Initial items creation and Delphi expert inquiry

A literature review, along with qualitative interviews, was conducted to develop the initial version (Version 1) of the questionnaire. The initial version of the questionnaire comprised 37 items, organized into four dimensions. The interviews involved 11 obstetric nurses and seven obstetric nursing managers. The 11 obstetric nurses interviewed were aged between 25 and 35 years. Four of them held intermediate-level titles, while the remaining seven had junior titles. One nurse had experience in the endocrinology department, and two had not received training on gestational diabetes mellitus (GDM). The seven obstetric nursing managers, aged 37 to 45 years, all held intermediate-level titles and had 4 to 9 years of experience in obstetric nursing management. Ten experts were selected to conduct two rounds of Delphi expert inquiry for the questionnaire, the degree of coordination and concentration of the first round of expert opinions is listed in Table 1. According to the first round of Delphi expert inquiry, the value of Kendall *W* is 0.254, the value of *x*^2^ is 91.264, and *p* (0.000) is less than 0.05. The CV values of items “I can provide referral guidance for newborns with abnormal blood sugars.” and “I can use multiple channels to encourage all pregnant women with gestational diabetes to participate in the self-management of their diabetes.” is 0.278 and 0.314, respectively, which are higher than the filtering criteria (less than 0.25), thus, these two items were deleted. The deleted items are not included in the table below. After the first round of correspondence, 35 items remain from the original list.

a) Professional knowledge dimension (KD)

**Table 1:** Degree of coordination and concentration of first round of expert opinions.

Items	Degree of coordination	Degree of concentration	
	CV	*Mj*	*Kj*
Professional knowledge dimension (KD)			
1. I am aware of the risk factors of GDM and its adverse effects on both the mother and the child	0.065	4.90	90
2. I am familiar with the diagnostic criteria and glycemic control goals for GDM	0.088	4.80	80
3. I am proficient in the exercise guidance knowledge of GDM	0.103	4.70	70
4. I am proficient in dietary guidance for GDM	0.065	4.90	90
5. I am familiar with the medication knowledge and adverse reactions of GDM	0.065	4.90	90
6. I am aware of the common complications of GDM (such as hypoglycemia, ketoacidosis, hypertonic coma, macrosomia, etc.)	0.088	4.80	80
7. I am aware of the normal range and clinical significance of blood glucose and glycosylated hemoglobin in GDM patients	0.065	4.90	90
8. I am aware of the timing, normal range and clinical significance of blood glucose monitoring in newborns born with GDM	0.065	4.90	90
9. I am aware of the clinical manifestations and nursing measures of neonatal hypoglycemia in GDM patients	0.065	4.90	90
10. I am aware of the content of postpartum health education and discharge review guidance for GDM	0.088	4.80	80
11. I can accurately assess the psychological characteristics of GDM patients and apply psychological knowledge to provide guidance	0.088	4.80	80
12. I am knowledgeable about relevant healthcare policies, regulations, and ethical standards	0.088	4.80	80
Professional skill dimension (SD)			
13. I can do health risk assessments for GDM patients	0.088	4.80	80
14. I am capable of providing preventive health guidance for pregnant women with high-risk factors for GDM	0.103	4.70	70
15. I can accurately assess whether the lifestyle of GDM patients is healthy	0.112	4.60	60
16. I am proficient in operating techniques (such as blood sugar monitoring, insulin injection, etc.) for GDM patients	0.065	4.90	90
17. I can master the monitoring skills of fetal heart and fetal movement in GDM patients	0.065	4.90	90
18. I can provide effective psychological intervention for patients with GDM (such as relieving negative emotions and alleviating excessive concerns about the safety of the fetus in the womb)	0.103	4.70	70
19. I can provide individualized dietary plans for GDM patients	0.065	4.90	90
20. I can assess whether patients with GDM have exercise contraindications and provide appropriate exercise guidance for those without contraindications	0.103	4.70	70
21. I can give proper medication guidance to GDM patients	0.088	4.80	80
22. I can teach GDM patients to self-assessment and self-management	0.103	4.70	70
23. I am able to implement emergency techniques for GDM patients in emergency situations (such as hypoglycemia, hypertonic coma)	0.065	4.90	90
Professional ability dimension (AbD)			
24. When implementing interventions for GDM patients, I can comprehensively analyze the data of GDM patients and quickly and effectively identify the care issues	0.088	4.80	80
25. I can combine my theoretical knowledge and practical experience to accept the opinions of relevant professionals critically	0.112	4.60	60
26. I am able to communicate effectively with GDM patients and their caregivers from different family backgrounds and cultural levels	0.103	4.70	70
27. I was able to find the problems that caused the miscommunication at work in time and put forward the improvement measures accordingly	0.103	4.70	70
28. I can give lectures on GDM health education	0.117	4.40	40
29. I can actively learn professional knowledge about GDM through literature reading and academic exchanges	0.103	4.70	70
30. I can conduct research or write research papers related to GDM	0.188	4.20	40
31. I can master the use of basic office software and collect and sort out the data of GDM patients	0.159	4.40	50
Personal attitude dimension (AtD)			
32. I can take the initiative to provide warm and considerate care to GDM patients	0.103	4.70	70
33. I am able to deal with the problems encountered in the management of GDM patients in a positive way	0.103	4.70	70
34. I have the spirit of being prudent and independent, and can carry out all kinds of nursing work regularly	0.103	4.70	70
35. I can use the spirit of “Nightingale” to guide myself to realize personal value in nursing post	0.117	4.40	40

One expert suggested amending “aware of” to “familiar with” in Item K1 to appropriately raise the standard for obstetric nurses; two experts suggested amending “am familiar with” to “know…very well” in Item K2; one expert suggested adding the words “mother and child” before “complications” in Item K6 to provide a clearer point of reference; two experts suggested amending “discharge review” to “review” in Item K10 to provide a more comprehensive statement; and one expert suggested amending Item K11 to read, “I possess the psychological knowledge required to assess the psychological characteristics of patients with GDM,” which focuses on competence, and is consistent with the professional knowledge aspect of the entry after the amendment. These suggestions were accepted.

b) Professional skill dimension (SD)

Three experts suggested amending “pregnant women” to “patients” in Item S14 to make the title more rigorous after unification; one expert suggested removing “whether…is healthy” from Item S15; one expert suggested amending Item S18 to “I can use psychological knowledge and skills to provide effective psychological intervention for GDM patients”; and one expert suggested that Item S19 be revised to “I can provide targeted dietary guidance for GDM patients.” One expert suggested that Item S20 be changed to “I can provide targeted exercise guidance to GDM patients”; one expert suggested that Item S22 be changed to “I can teach GDM patients how to self-assess and manage their blood glucose levels.” These suggestions were accepted.

c) Professional ability dimension (AbD)

One expert suggested adding an item “I can teach GDM patients how to perform emergency self-rescue measures in the event of hypoglycemia”; one expert suggested adding an item “I can teach GDM patients to recognize the early symptoms of diabetic ketoacidosis”; one expert suggested adding “optimal” before the Item Ab24 “care issues” and modifying it; one expert suggested modifying the Item Ab31 “basic” to “commonly used.” These suggestions were accepted.

d) Personal attitude dimension (AtD)

One expert suggested that the target audience for Item At32 should be added and amended to “I can take the initiative to provide warm and considerate care to GDM patients and their families.”

The two items added based on expert opinions fall under the professional competence dimension. According to the results of the first round of consultations, the order and number of items in the professional knowledge and professional skills dimensions remain unchanged. The order of items in the professional competence and personal attitude dimensions has been adjusted after reorganization, as shown in Table 2.

**Table 2:** Items of professional ability and personal attitude dimension after first round of expert opinions.

Dimension	Items
Professional ability (AbD)	24. I can teach GDM patients how to perform emergency self-rescue measures in the event of hypoglycemia
	25. I can teach GDM patients to recognize the early symptoms of diabetic ketoacidosis
	26. When implementing interventions for GDM patients, I can comprehensively analyze the data of GDM patients and quickly and effectively identify the optimal care issues
	27. I can combine my theoretical knowledge and practical experience to accept the opinions of relevant professionals critically
	28. I am able to communicate effectively with GDM patients and their caregivers from different family backgrounds and cultural levels
	29. I was able to find the problems that caused the miscommunication at work in time and put forward the improvement measures accordingly
	30. I can give lectures on GDM health education
	31. I can actively learn professional knowledge about GDM through literature reading and academic exchanges
	32. I can conduct research or write research papers related to GDM.
	33. I can master the use of commonly used office software and collect and sort out the data of GDM patients
Personal attitude (AtD)	34. I can take the initiative to provide warm and considerate care to GDM patients and their families
	35. I am able to deal with the problems encountered in the management of GDM patients in a positive way
	36. I have the spirit of being prudent and independent, and can carry out all kinds of nursing work regularly
	37. I can use the spirit of “Nightingale” to guide myself to realize personal value in nursing post

In the second round of Delphi expert inquiry, the value of Kendall *W* is 0.164, the value of *x*^2^ is 58.90, and *p* (0.000) is less than 0.05. The CV value for each item was less than 0.25, and the mean value for each item was higher than 3.5. The second round of expert consultations ended without further constructive comments. A total of 37 questionnaire items were consolidated with the experts’ opinions to form the initial questionnaire, which will be administered in the next step.

### 3.2 Phase 2: evaluation of the questionnaire

#### 3.2.1 Pilot study

Thirty obstetric nurses from three hospitals in Luoyang City were selected by convenience sampling method in April 2022 to pre-survey the second version of the questionnaire to test the suitability of each item for obstetric nurses. All 30 obstetric nurses surveyed found the questionnaire items to be easy understand, convenient to fill out, and acceptable in terms of time. No further changes were made to the questionnaire items to create a testing version.

#### 3.2.2 Testing of validity and reliability

##### 3.2.2.1 Demographic characteristics of participant

A total of 239 questionnaires were analyzed, the characteristics of these participants are shown in Table 3.

**Table 3:** Demographic characteristics of participants in the validation study.

Descriptions	No. of participants	Proportions (%)
Gender		
Female	239	100.00
Male	0	0
Age		
≤28	118	49.37
29–35	111	46.44
≥36	10	4.18
Ethnic		
Han	222	92.89
Minorities	17	7.11
Marital status		
Married	150	62.76
Unmarried	89	37.24
Divorced or otherwise	0	0
Delivery history		
Yes	87	36.40
No	152	63.60
Education		
Secondary school	6	2.51
College	75	31.38
Bachelor	158	66.11
Professional title		
Nurse	26	10.88
Senior nurse	158	66.11
Supervisor nurse	55	23.01
Years of work in obstetric care		
1–5	146	61.09
6–10	75	31.38
≥11	18	7.53
Work experience in endocrinology		
Yes	8	3.35
No	231	96.65
Number of training sessions on gestational diabetes		
None	28	11.72
Once/year	108	45.61
2–3 times/year	92	38.49
≥4 times/year	10	4.18

##### 3.2.2.2 Project analysis

a) Critical ratio method

The collected valid questionnaires were analyzed using the proximity ratios, and the results are presented in Table 4; the CR values for all entries were >0.3 and *p* was <0.01; thus, all items were retained.

b) Correlation analysis method

**Table 4:** Critical ratio analysis of questionnaire (*n* = 239).

Items	CR	*p*	Results	Items	CR	*p*	Results
1	14.874	0.000^**^	Remain	20	10.938	0.000^**^	Remain
2	13.811	0.000^**^	Remain	21	11.517	0.000^**^	Remain
3	10.891	0.000^**^	Remain	22	12.733	0.000^**^	Remain
4	15.331	0.000^**^	Remain	23	23.163	0.000^**^	Remain
5	18.390	0.000^**^	Remain	24	3.439	0.001^**^	Remain
6	17.555	0.000^**^	Remain	25	4.556	0.000^**^	Remain
7	16.911	0.000^**^	Remain	26	19.899	0.000^**^	Remain
8	13.824	0.000^**^	Remain	27	13.166	0.000^**^	Remain
9	15.744	0.000^**^	Remain	28	11.918	0.000^**^	Remain
10	14.923	0.000^**^	Remain	29	19.137	0.000^**^	Remain
11	11.236	0.000^**^	Remain	30	10.028	0.000^**^	Remain
12	10.757	0.000^**^	Remain	31	12.070	0.000^**^	Remain
13	15.853	0.000^**^	Remain	32	10.652	0.000^**^	Remain
14	16.864	0.000^**^	Remain	33	11.703	0.000^**^	Remain
15	12.302	0.000^**^	Remain	34	9.399	0.000^**^	Remain
16	10.289	0.000^**^	Remain	35	11.482	0.000^**^	Remain
17	15.022	0.000^**^	Remain	36	9.475	0.000^**^	Remain
18	15.372	0.000^**^	Remain	37	7.282	0.000^**^	Remain
19	13.988	0.000^**^	Remain				

^**^The values of *p* were significantly correlated at the 0.01 level (bilaterally), the same as below.

The correlation between each item and the total score of the questionnaire was statistically significant (*p* < 0.01), in which the *r* of items Ab24 and Ab25 were less than 0.4, and the correlation with the total score was poor, and the r value of questionnaire item Ab24 “I can teach GDM patients to grasp the emergency self-rescue measures in the event of hypoglycemia” was 0.202. The r value of item Ab25, “I can teach GDM patients to recognize early symptoms of ketoacidosis,” was 0.269, and that of “I can teach GDM patients to recognize the early symptoms of ketoacidosis,” was 0.269, and these two items were deleted. The results of the homogeneity test analysis of the questionnaire are summarized in Table 5, and the handling procedures of missing data can be seen in Tables 4, 5 and Figure 1.

c) Cronbach’s *α* coefficient method

**Table 5:** Homogeneity test analysis of questionnaire.

Items	Correlation coefficient	*p*	Results	Items	Correlation coefficient	*p*	Results
1	0.729	0.000^**^	Remain	20	0.672	0.000^**^	Remain
2	0.701	0.000^**^	Remain	21	0.689	0.000^**^	Remain
3	0.608	0.000^**^	Remain	22	0.757	0.000^**^	Remain
4	0.739	0.000^**^	Remain	23	0.826	0.000^**^	Remain
5	0.749	0.000^**^	Remain	24	0.202	0.002^**^	Delete
6	0.779	0.000^**^	Remain	25	0.269	0.000^**^	Delete
7	0.704	0.000^**^	Remain	26	0.834	0.000^**^	Remain
8	0.712	0.000^**^	Remain	27	0.736	0.000^**^	Remain
9	0.677	0.000^**^	Remain	28	0.676	0.000^**^	Remain
10	0.732	0.000^**^	Remain	29	0.826	0.000^**^	Remain
11	0.671	0.000^**^	Remain	30	0.677	0.000^**^	Remain
12	0.687	0.000^**^	Remain	31	0.641	0.000^**^	Remain
13	0.795	0.000^**^	Remain	32	0.668	0.000^**^	Remain
14	0.803	0.000^**^	Remain	33	0.719	0.000^**^	Remain
15	0.722	0.000^**^	Remain	34	0.567	0.000^**^	Remain
16	0.598	0.000^**^	Remain	35	0.712	0.000^**^	Remain
17	0.703	0.000^**^	Remain	36	0.567	0.000^**^	Remain
18	0.757	0.000^**^	Remain	37	0.516	0.000^**^	Remain
19	0.754	0.000^**^	Remain				

**Figure 2:**
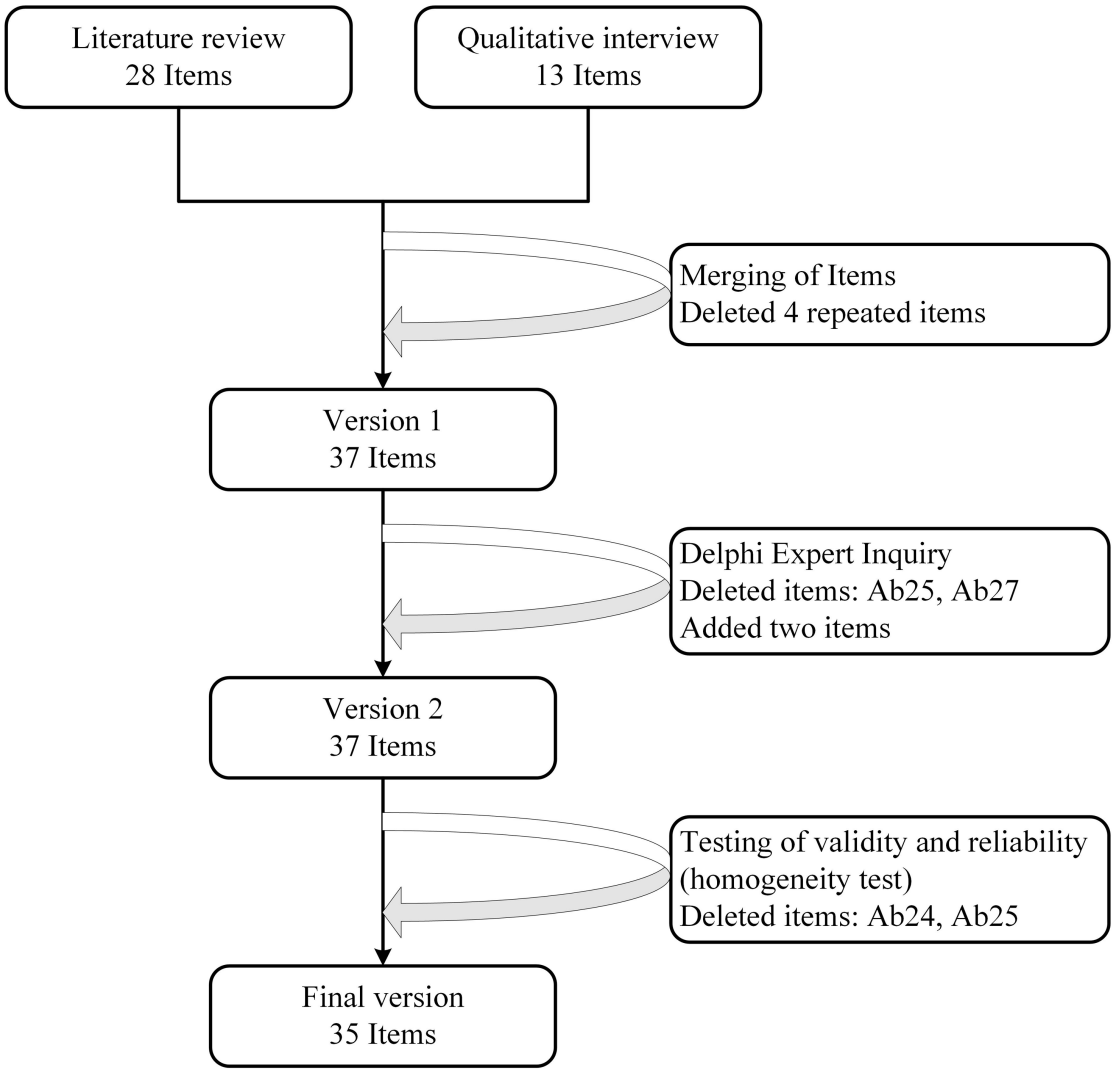
Flow diagram of item reduction process.

The results in Table 6 show that the overall Cronbach’s *α* value of the initial questionnaire was 0.970, and the Cronbach’s *α* value did not increase after the deletion of question items, so there is no need to delete all the questions for the time being (see Figure 2).

**Table 6:** Questionnaire Cronbach’s *α* analysis form.

Items	Cronbach’s *α* value for items deleted	Items	Cronbach’s *α* value for items deleted
1	0.969	19	0.969
2	0.969	20	0.970
3	0.970	21	0.969
4	0.969	22	0.969
5	0.969	23	0.969
6	0.969	24	0.969
7	0.969	25	0.969
8	0.969	26	0.970
9	0.970	27	0.969
10	0.969	28	0.970
11	0.970	29	0.970
12	0.970	30	0.970
13	0.969	31	0.969
14	0.969	32	0.970
15	0.969	33	0.969
16	0.970	34	0.970
17	0.969	35	0.970
18	0.969		

##### 3.2.2.3 Validity analysis

a) Content validity

A total of five experts were invited to score this study, and the calculated S-CVI/Ave was 0.95, and the I-CVI was 0.80–1.00. It met the reference standard of S-CVI/Ave ≥0.90 and I-CVI ≥0.78. The scoring results are shown in Table 7.

b) Structural validity

**Table 7:** Score of expert content validity (*n* = 5).

Item	Number of experts with rating of ≥3	I-CVI	Item	Number of experts with rating of ≥3	I-CVI
1	5	1.0	19	5	1.0
2	5	1.0	20	5	1.0
3	5	1.0	21	5	1.0
4	5	1.0	22	5	1.0
5	5	1.0	23	5	1.0
6	5	1.0	24	5	1.0
7	5	1.0	25	5	1.0
8	5	1.0	26	5	1.0
9	5	1.0	27	5	1.0
10	5	1.0	28	4	0.8
11	4	0.8	29	5	1.0
12	5	1.0	30	4	0.8
13	5	1.0	31	4	0.8
14	5	1.0	32	5	1.0
15	5	1.0	33	5	1.0
16	5	1.0	34	5	1.0
17	5	1.0	35	4	0.8
18	5	1.0			

The Kaiser-Meyer-Olkin (KMO) measure is 0.814, and Bartlett’s test of sphericity is significant (*χ*^2^ = 5470.775, df = 328, *p* < 0.001), indicating the data is suitable for factor analysis. Principal component analysis with varimax rotation was applied, which identified four principal components with eigenvalues exceeding 1.0, collectively explaining 73.45% of the total variance. All items demonstrated strong factor loading, ranging from 0.601 to 0.929. The emergent factor structure showed strong consistency with the hypothetical framework, aligning well with the original conceptual dimensions. The factor loading matrix is shown in Table 8.

**Table 8:** Factor load matrix diagram.

Items	Factor 1	Factor 2	Factor 3	Factor 4	Common factor variance
28	0.869				0.847
30	0.856				0.823
25	0.850				0.916
24	0.847				0.801
29	0.812				0.842
31	0.808				0.854
27	0.737				0.921
26	0.636				0.823
8		0.736			0.847
5		0.733			0.814
7		0.729			0.808
11		0.721			0.906
4		0.694			0.846
10		0.686			0.845
3		0.668			0.838
9		0.648			0.919
1		0.637			0.912
12		0.628			0.861
6		0.619			0.809
2		0.607			0.819
17			0.929		0.801
20			0.908		0.901
19			0.878		0.850
16			0.833		0.803
23			0.812		0.809
15			0.794		0.814
13			0.765		0.901
22			0.702		0.842
14			0.698		0.882
21			0.636		0.812
18			0.601		0.906
34				0.918	0.843
35				0.892	0.897
32				0.808	0.852
33				0.794	0.803

The structural validity of the questionnaire was assessed using CFA with AMOS 23.0. The model exhibited acceptable fit indices, indicating that the proposed factor structure adequately represented the data: *χ*^2^/df = 2.39, GFI = 0.81, AGFI = 0.80, RMSEA = 0.08, TLI = 0.90, IFI = 0.92, CFI = 0.92. These indices collectively support the adequacy of the model fit, suggesting that the factor structure of the questionnaire is well-suited to the data (34, 35).

The Pearson correlation coefficients between dimensions ranged from 0.632 to 0.903, and the Pearson correlation coefficients between dimensions and the total questionnaire ranged from 0.754 to 0.967, which were all greater than 0.7, suggesting a strong correlation. See Table 9 for details.

**Table 9:** Correlation between each dimension and the dimension and the questionnaire (Pearson correlation coefficient).

Dimension	Professional knowledge	Professional skill	Professional ability	Personal attitude	Total
Professional knowledge	1.00				
Professional skill	0.903^**^	1.00			
Professional ability	0.818^**^	0.849^**^	1.00		
Personal attitude	0.656^**^	0.703^**^	0.632^**^	1.00	
Total	0.956^**^	0.967^**^	0.920^**^	0.754^**^	1.00

##### 3.2.2.4 Reliability analysis

a) Internal consistency reliability

The Cronbach’s *α* coefficients of the four dimensions were in the range of 0.793–0.928, and the Cronbach’s *α* coefficient of the total questionnaire was 0.970.

b) Folded half reliability

The fold-half reliability of the total questionnaire is 0.950, and the fold-half coefficients of the four dimensions are 0.787–0.938.

c) Retest reliability

The retest reliability coefficients of the four dimensions are in the range of 0.899–0.964, and the retest reliability coefficient of the total questionnaire is 0.907. See Table 10 for details.

**Table 10:** Test results of reliability of each dimension and total score of questionnaire.

Dimension	Cronbach’s *α* coefficient	Half-confidence	Re-testing reliability
Professional knowledge	0.924	0.928	0.917^**^
Professional skill	0.928	0.938	0.939^**^
Professional ability	0.908	0.837	0.964^**^
Personal attitude	0.793	0.787	0.899^**^
Overall level of the questionnaire	0.970	0.950	0.907^**^

## 4 Discussion

### 4.1 Necessity of questionnaire development

Due to the fact that GDM increases perinatal complications and also contributes to an increased risk of distant morbidity of diseases such as type 2 diabetes in the mother and offspring, the competence of obstetric nurses in managing patients with gestational diabetes affects the management of maternal glycemia both in the hospital and after discharge.

A review of the literature revealed the existence of instruments such as the questionnaire for the health education needs of GDM patients and the questionnaire for GDM self-management ability, both of which focus on the patient perspective. However, no instrument appears to be specifically designed to assess the competence of obstetric nurses in the management of GDM, which complicates efforts to evaluate the current state of practice in this area. The aim of this study was to develop an appropriate questionnaire to provide a tool to measure the competence of obstetric nurses in the management of GDM.

### 4.2 Reliability and validity testing of the questionnaire

#### 4.2.1 Reliability evaluation

Reliability refers to the consistency and dependability of a measurement tool, representing the reliability of the assessment instrument. Generally, a Cronbach’s *α* coefficient between 0.70 and 0.80 indicates acceptable internal consistency reliability, 0.80 and 0.90 indicates high internal consistency reliability, and a Cronbach’s *α* coefficient ≥0.90 indicates very high internal consistency reliability. Split-half reliability is considered acceptable with a correlation coefficient greater than 0.70. Test-retest reliability greater than 0.70 indicates good stability and consistency of the questionnaire (36).

In this study’s reliability testing, the Cronbach’s *α* coefficients for the various dimensions ranged from 0.793 to 0.928, with the overall questionnaire having a Cronbach’s *α* coefficient of 0.970. The split-half coefficients for the dimensions ranged from 0.787 to 0.938, with the overall questionnaire having a split-half reliability of 0.950. The test-retest reliability coefficients for the dimensions ranged from 0.899 to 0.964, with the overall questionnaire having a test-retest reliability coefficient of 0.907. No items were deleted during the analysis.

#### 4.2.2 Validity evaluation

The validity of the “Questionnaire on the Management Ability of Obstetric Nurses for Patients with Gestational Diabetes Mellitus” was evaluated using structural validity and content validity. The questionnaire was designed based on the theory of job competence, with clear structures for each dimension. Pearson correlation coefficients were used for analysis, where higher coefficients indicate a stronger association between variables. Generally, a coefficient between 0.8 and 1.0 represents a very strong correlation, while a coefficient between 0.6 and 0.8 represents a strong correlation. The results showed that the correlation coefficients between the dimensions and the overall questionnaire ranged from 0.754 to 0.967, and the correlation coefficients between the dimensions ranged from 0.632 to 0.903. This indicates that the structure of the dimensions is reasonable and reliable.

Content validity reflects the appropriateness and representativeness of the questionnaire items. The questionnaire items were developed through literature review, qualitative interviews, expert consultations, and pilot surveys, ensuring they accurately reflect the needs of obstetric nurses in managing GDM patients. Generally, a questionnaire is considered to meet standards if the I-CVI is ≥0.78 and the S-CVI/Ave is ≥0.8. In this study, five experts in relevant fields evaluated the relevance of each item and the questionnaire as a whole. The I-CVI ranged from 0.80 to 1.00, and the S-CVI/Ave was 0.95, indicating good content validity, suggesting that the questionnaire can effectively assess the management ability of obstetric nurses for GDM.

Based on the above data analysis, it can be concluded that the questionnaire meets the psychometric standards for questionnaire development and has good reliability and validity.

### 4.3 Scientificity and applicability of the questionnaire

This study, guided by the theory of job competence, initially developed a pool of 37 items through a literature review and semi-structured interviews. The content of the questionnaire was modified and refined through two rounds of Delphi expert inquiry. The final items were determined after the research team had discussed them. To ensure the applicability and acceptability of the questionnaire, a pilot survey was conducted with 30 obstetric nurses. Following reliability and validity testing, the final questionnaire, consisting of 35 items, was established, see details in Supplementary Data Sheet 1. The development process of this questionnaire was scientific and reasonable.

The time required for obstetric nurses to complete this questionnaire is less than 10 min. According to feedback, the content of the questionnaire items is easy to understand, convenient to fill out, and takes a short amount of time, indicating good applicability.

### 4.4 Implementation challenges and training requirement

#### 4.4.1 Implementation challenges

Potential implementation challenges in real-world clinical settings must also be considered.

1) Psychological barriers to change.

In clinical settings, nurses and healthcare workers may resist adopting new tools due to their reliance on established practices or skepticism regarding the tool’s effectiveness. This resistance is particularly prevalent in busy hospitals where nurses may hesitate to invest time in learning new tools. To overcome this barrier, healthcare institutions should emphasize the value of the new tool, provide regular training, and demonstrate its impact on improving GDM management and patient education. Collecting regular feedback from nurses and making necessary adjustments to the tool can also help reduce resistance.

2) Resource limitations.

The implementation of the questionnaire may be hindered by resource limitations, including insufficient time, staff, and technical support. In busy clinical settings, heavy nurse workloads, especially in understaffed environments, can make it difficult to complete the questionnaire. A lack of administrative and technical support may also hinder integration into workflows. To address this, hospitals should allocate dedicated time for nurses to complete the questionnaire and receive training. Using digital platforms can streamline the process. Data should be systematically analyzed to provide actionable feedback for decision-making and improvements, supported by professional expertise and adequate resources.

3) Variability in nurse training and experience.

Differences in nurses’ training and experience in GDM management can affect the accuracy and effectiveness of the questionnaire. Inexperienced nurses may misinterpret certain items, leading to skewed results. To minimize this, hospitals should implement standardized GDM management training programs and regularly assess nurses’ competencies. This ensures consistent, accurate use of the tool across the clinical setting.

4) Sustained use and long-term commitment.

Successful implementation of the questionnaire depends not only on its initial application but also on its sustained use over time. Nurses may lose interest, and hospital management may neglect its ongoing use due to increasing workload pressures. To ensure continued relevance, regular evaluations and updates should be conducted, incorporating feedback from nurses and other stakeholders. This will ensure the tool remains aligned with evolving GDM management guidelines and continues to provide value in clinical practice.

These barriers highlight the challenges of effectively implementing the questionnaire in clinical settings. Addressing them with targeted strategies will help ensure the tool’s long-term success and impact.

#### 4.4.2 Training requirement

To ensure the successful implementation of the questionnaire tool, comprehensive nurse training is essential. The training should emphasize the purpose and significance of the questionnaire, clarifying its role in assessing GDM management. It should also foster nurses’ motivation to use the tool effectively. Additionally, the training must provide detailed explanations of the questionnaire’s structure and the meaning of each item, reducing the risk of misunderstandings that could compromise data quality. Nurses should be proficient in using both the paper and electronic versions of the questionnaire. Training methods should be adaptable: face-to-face training is ideal in the initial stages, allowing for interactive discussions, while online training offers flexibility for busy nurses. To accommodate workloads, training sessions should be scheduled during less busy periods, and annual refresher courses should be held to update knowledge and reinforce skills. Moreover, hospitals should establish assessment and feedback mechanisms to gauge the effectiveness of the training, and provide ongoing support to help nurses address any challenges. This approach will not only enhance nurses’ competence but also contribute to better GDM management and improved patient outcomes.

### 4.5 Limitations

This study has several limitations that should be considered when interpreting the findings. Firstly, while the expert panel was well-qualified, the selection process may have introduced biases, as the panel consisted primarily of highly experienced professionals. This may not fully represent the views of nurses with less experience in GDM management. Secondly, the cultural context in which the questionnaire was developed may limit its applicability in settings with different healthcare systems or cultural practices. The tool was based on national guidelines of China, but future studies should examine its cross-cultural applicability and validate it in diverse healthcare environments. The translation and back-translation process was not conducted due to the limited scope of the study, which was primarily focused on the development and validation of the questionnaire within a Chinese obstetric nursing population. As the primary aim of this study was to assess the questionnaire’s psychometric properties in a Chinese cultural context, translation into English and subsequent back-translation were not deemed necessary at this stage. However, future studies are recommended to explore the cross-cultural adaptation and validation of the questionnaire for use in international settings. Thirdly, the sample size in the pilot study may be considered small for initial testing, potentially limiting the external validity of the results. A larger and more diverse sample would enhance the generalizability of the questionnaire across different populations of obstetric nurses. Future research should explore the broader implementation of the questionnaire, including its utility in different clinical settings and its impact on improving nursing practices in GDM management.

## 5 Conclusion

1) This study developed an assessment tool for evaluating the management ability of obstetric nurses for GDM patients. The questionnaire consists of four dimensions and 35 items, reflecting the management ability of obstetric nurses for GDM patients. The dimensions and items demonstrate the content of the competence theory. The questionnaire shows good stability and reliability and validity.
2) The questionnaire has good applicability, with content that is easy to understand and items that are simple and easy to comprehend. It can be used by obstetric nurses with different levels of education and serves as a reliable assessment tool for evaluating the management ability of obstetric nurses for GDM patients.

## Data Availability

The original contributions presented in the study are included in the article/Supplementary material, further inquiries can be directed to the corresponding authors.

## References

[1] McIntyre HD Catalano P Zhang C Desoye G Mathiesen ER Damm P. Gestational diabetes mellitus. Nat Rev Dis Primers. (2019) 5:47. doi: 10.1038/s41572-019-0098-8, PMID: 31296866

[2] Sweeting A Wong J Murphy HR Ross GP. A clinical update on gestational diabetes mellitus. Endocr Rev. (2022) 43:763–93. doi: 10.1210/endrev/bnac003, PMID: 35041752 PMC9512153

[3] Johns EC Denison FC Norman JE Reynolds RM. Gestational diabetes mellitus: mechanisms, treatment, and complications. Trends Endocrinol Metab. (2018) 29:743–54. doi: 10.1016/j.tem.2018.09.004 30297319

[4] Chiefari E Arcidiacono B Foti D Brunetti A. Gestational diabetes mellitus: an updated overview. J Endocrinol Investig. (2017) 40:899–909. doi: 10.1007/s40618-016-0607-5, PMID: 28283913

[5] International Diabetes Federation. IDF diabetes atlas. 10th ed. Brussels: International Diabetes Federation (2021).

[6] Wei Y Juan J Yang H. Comprehensive management of gestational diabetes mellitus in China. Matern-Fetal Med. (2021) 3:161–3. doi: 10.1097/FM9.0000000000000113

[7] Zhu H Zhao Z Xu J Chen Y Zhu Q Zhou L. The prevalence of gestational diabetes mellitus before and after the implementation of the universal two-child policy in China. Front Endocrinol. (2022) 13:960877. doi: 10.3389/fendo.2022.960877, PMID: 36060951 PMC9433653

[8] Buchanan TA Xiang AH Page KA. Gestational diabetes mellitus: risks and management during and after pregnancy. Nat Rev Endocrinol. (2012) 8:639–49. doi: 10.1038/nrendo.2012.96, PMID: 22751341 PMC4404707

[9] Duarte-Gardea MO Gonzales-Pacheco DM Reader DM Thomas AM Wang SR Gregory RP. Academy of nutrition and dietetics gestational diabetes evidence-based nutrition practice guideline. J Acad Nutr Diet. (2018) 118:1719–42. doi: 10.1016/j.jand.2018.03.014, PMID: 29859757

[10] Zhang M Zhou Y Zhong J Wang K Ding Y Li L. Current guidelines on the management of gestational diabetes mellitus: a content analysis and appraisal. BMC Pregnancy Childbirth. (2019) 19:200–15. doi: 10.1186/s12884-019-2343-2, PMID: 31196116 PMC6567433

[11] Mensah GP ten Ham-Baloyi W van Rooyen D Jardien-Baboo S. Guidelines for the nursing management of gestational diabetes mellitus: an integrative literature review. Nurs Open. (2020) 7:78–90. doi: 10.1002/nop2.324, PMID: 31871693 PMC6918019

[12] Schellinger MM Abernathy MP Amerman B May C Foxlow LA Carter AL. Improved outcomes for Hispanic women with gestational diabetes using the Centering Pregnancy^©^ group prenatal care model. Matern Child Health J. (2017) 21:297–305. doi: 10.1007/s10995-016-2114-x, PMID: 27423239

[13] National Collaborating Centre for Women’s and Children’s Health. Diabetes in pregnancy: management of diabetes and its complications from preconception to the postnatal period. London: RCOG Press (2008). 21370515

[14] Yang Z Li Z Chen H Wang Z. The differences of gestational diabetes outpatient management between China and the United Kingdom: from the perspective of a first-year resident. Matern-Fetal Med. (2021) 3:164–8. doi: 10.1097/FM9.0000000000000110

[15] Juan J Yang H. Prevalence, prevention, and lifestyle intervention of gestational diabetes mellitus in China. Int J Environ Res Public Health. (2020) 17:9517. doi: 10.3390/ijerph17249517, PMID: 33353136 PMC7766930

[16] Xu Z Xia H. A qualitative exploration of a nurse practitioner role in gestational diabetes mellitus management. J Nurse Pract. (2023) 19:104632. doi: 10.1016/j.nurpra.2023.104632

[17] Li L Bin Z Yan D. Practice and effect evaluation of gestational diabetes mellitus specialist practice model. Chin J Nurs. (2017) 8:313. doi: 10.11648/j.ajns.20190806.14

[18] Choi ES Oh JA Park CS. A study of nurses’ knowledges on gestational diabetes mellitus. Korean J Women Health Nurs. (2001) 7:419–31. doi: 10.4069/kjwhn.2001.7.4.419

[19] Lakshmi K. Effectiveness of nursing care of antenatal mothers with gestational diabetes mellitus. Asian J Nurs Educ Res. (2020) 10:286–90. doi: 10.5958/2349-2996.2020.00060.9

[20] Pujiyanto TI Wulaningsih I. Nurses’ role in taking care of gestational diabetes mellitus patients: a qualitative study. J Ners. (2021) 16:162–8. doi: 10.20473/jn.v16i2.29811

[21] Mrayyan MT Abunab HY Khait AA Rababa MJ Al-Rawashdeh S Algunmeeyn A. Competency in nursing practice: a concept analysis. BMJ Open. (2023) 13:e067352. doi: 10.1136/bmjopen-2022-067352, PMID: 37263688 PMC10255110

[22] Fukada M. Nursing competency: definition, structure and development. Yonago Acta Med. (2018) 61:001–7. doi: 10.33160/yam.2018.03.001, PMID: 29599616 PMC5871720

[23] Neary M. Curriculum studies in post-compulsory and adult education: a study guide for teachers and student teachers. Cheltenham: Nelson Thornes (2002).

[24] Giorgi A. The theory, practice, and evaluation of the phenomenological method as a qualitative research procedure. J Phenomenol Psychol. (1997) 28:235–60. doi: 10.1163/156916297X00103

[25] Falzarano M Zipp GP. Seeking consensus through the use of the Delphi technique in health sciences research. J Allied Health. (2013) 42:99–105. PMID: 23752237

[26] Niederberger M Spranger J. Delphi technique in health sciences: a map. Front Public Health. (2020) 8:457. doi: 10.3389/fpubh.2020.00457, PMID: 33072683 PMC7536299

[27] de Villiers MR de Villiers PJ Kent AP. The Delphi technique in health sciences education research. Med Teach. (2005) 27:639–43. doi: 10.1080/13611260500069947 16332558

[28] Almanasreh E Moles R Chen TF. Evaluation of methods used for estimating content validity. Res Soc Adm Pharm. (2019) 15:214–21. doi: 10.1016/j.sapharm.2018.03.066, PMID: 29606610

[29] Qian J. Development and reliability and validity of health education demand questionnaire for patients with gestational diabetes mellitus In: Master thesis. Hefei: Anhui Medical University (2020)

[30] Oksenberg L Kalton G. New strategies for pretesting survey questions. J Off Stat. (1991) 7:349.

[31] Watson R Thompson DR. Use of factor analysis in journal of advanced nursing: literature review. J Adv Nurs. (2006) 55:330–41. doi: 10.1111/j.1365-2648.2006.03915.x 16866827

[32] Conway JM Huffcutt AI. A review and evaluation of exploratory factor analysis practices in organizational research. Organ Res Methods. (2003) 6:147–68. doi: 10.1177/1094428103251541

[33] Barbaranelli C Lee CS Vellone E Riegel B. The problem with Cronbach’s alpha: Comment on Sijtsma and Van der Ark (2015). Nurs Res. (2015) 64:140–5. doi: 10.1097/NNR.0000000000000079, PMID: 25738626 PMC4351779

[34] Sun Z. (2018). Medical statistics. 3 Beijing: People’s Medical Publishing House. 369–381.

[35] Wu M. (2019). Structural equation modeling: AMOS operations and applications. Chongqing: Chongqing University Press. 212–240.

[36] Stanton JM Sinar EF Balzer WK Smith PC. Issues and strategies for reducing the length of self-report scales. Pers Psychol. (2002) 55:167–94. doi: 10.1111/j.1744-6570.2002.tb00108.x

