# Supplementary material for: Development of a questionnaire to evaluate the management of gestational diabetes mellitus patients among obstetric nurses
Source: Front Public Health. 2025 Apr 23;13:1521673. doi: 10.3389/fpubh.2025.1521673 (PMC12055769; doi:10.3389/fpubh.2025.1521673)
Supplement: Supplementary file 1 [file Table_1.doc]

**Table S1** Outlines of interviews for obstetric nurses and obstetric nursing managers

| **Interviewee** | **Interview questions** |
| --- | --- |
| Obstetric nurses | 1. What do you think should be done to effectively manage GDM patients? |
|  | 2. What difficulties have you encountered in managing GDM patients, and how did you resolve them? |
|  | 3. What issues do you think you have in your management of GDM patients? |
|  | 4. In the management of GDM patients, what else do you need to learn more to meet the needs of the work? |
| Obstetric nursing managers | 1. What abilities do you think obstetric nurses should possess to effectively manage GDM patients? |
|  | 2. What abilities do you think obstetric nurses generally possess in the management of GDM patients? |
|  | 3. What abilities do you think obstetric nurses currently lack in the management of GDM patients? In what areas can they improve to enhance their management capabilities? |
