# Supplementary material for: Development of a questionnaire to evaluate the management of gestational diabetes mellitus patients among obstetric nurses
Source: Front Public Health. 2025 Apr 23;13:1521673. doi: 10.3389/fpubh.2025.1521673 (PMC12055769; doi:10.3389/fpubh.2025.1521673)
Supplement: Supplementary file 2 [file Data_Sheet_1.docx]

产科护士的妊娠期糖尿病患者管理能力评估问卷

亲爱的护理同仁：

这是一份关于产科护士妊娠期糖尿病患者管理能力的调查问卷，主要目的是了解产科护士对妊娠期糖尿病的护理现状。您的参与是完全自愿的，您在任何时候有权拒绝参与或退出调查，而不会受到任何惩罚或产生任何负面后果。如果您同意参加，请您依照自己的情况填答！您所填的各项资料只作为学术上统计分析使用，不会给您带来任何负面影响，参加研究的所有个人信息，我们保证为您保密，请您消除顾虑！

十分感谢您的合作，祝您生活愉快！

河南科技大学护理学院

朱永燕

**第一部分：产科护士一般资料**

1.性别： ○女 ○男

2.年龄： 岁

3.民族： ○汉族 ○少数民族

4.婚姻状况： ○已婚 ○未婚 ○离异或其他

5.分娩史： ○有 ○无

6.最高学历： ○中专 ○大专 ○本科 ○硕士研究生及以上

7.职称： ○护士 ○护师 ○主管护师 ○副主任护师及以上

8.产科护理工作年限： 年

9.有无内分泌科工作经历： ○有 ○无

10.接受妊娠期糖尿病相关培训次数： ○无 ○1次/年 ○2-3次/年 ○>3次/年

（培训计入标准：培训者为医生、营养师或主管护师以上护理人员，每次培训时间不少于30分钟）

1. **《产科护士的妊娠期糖尿病患者管理能力问卷》**

请根据您的真实情况在每一项对应的方框内划“√”。

| **维度** | **条目** | **能力分级** | | | | |
| --- | --- | --- | --- | --- | --- | --- |
|  |  | **完全没有能力** | **能力较差** | **能力一般** | **能力较强** | **很有能力** |
| **专业知识** | 1.我熟知妊娠期糖尿病的危险因素及对母儿的危害 |  |  |  |  |  |
|  | 2.我熟知妊娠期糖尿病的诊断标准及血糖控制目标 |  |  |  |  |  |
|  | 3.我熟练掌握妊娠期糖尿病的运动指导知识 |  |  |  |  |  |
|  | 4.我熟练掌握妊娠期糖尿病的饮食指导知识 |  |  |  |  |  |
|  | 5.我熟知妊娠期糖尿病的用药知识及药物的不良反应 |  |  |  |  |  |
|  | 6.我知晓妊娠期糖尿病母婴的常见并发症（如低血糖、酮症酸中毒、高渗性昏迷、巨大儿等） |  |  |  |  |  |
|  | 7.我知晓妊娠期糖尿病患者血糖、糖化血红蛋白的正常范围及临床意义 |  |  |  |  |  |
|  | 8.我知晓妊娠期糖尿病患者所产新生儿的血糖监测时机、正常范围及临床意义 |  |  |  |  |  |
|  | 9.我知晓妊娠期糖尿病患者所产新生儿低血糖的临床表现及护理措施 |  |  |  |  |  |
|  | 10.我知晓妊娠期糖尿病产后健康教育内容及出院指导知识 |  |  |  |  |  |
|  | 11.我具备评估妊娠期糖尿病患者心理特点的心理学知识 |  |  |  |  |  |
|  | 12.我了解相关医疗卫生政策法规及伦理规范 |  |  |  |  |  |
| **专业技能** | 13.我能对妊娠期糖尿病患者进行健康风险评估 |  |  |  |  |  |
|  | 14.我能对存在高危因素的妊娠期糖尿病患者进行预防性的保健指导 |  |  |  |  |  |
|  | 15.我能正确评估妊娠期糖尿病患者的生活方式 |  |  |  |  |  |
|  | 16.我能熟练掌握妊娠期糖尿病患者的血糖监测、胰岛素注射等操作技术 |  |  |  |  |  |
|  | 17.我能掌握妊娠期糖尿病患者胎心胎动的监测技能 |  |  |  |  |  |
|  | 18..我能运用心理学知识和技能对妊娠期糖尿病患者进行有效的心理干预（如疏导不良情绪、减轻对胎儿宫内安危的过度担忧） |  |  |  |  |  |
|  | 19.我能对妊娠期糖尿病患者进行针对性的饮食指导 |  |  |  |  |  |
|  | 20.我能对妊娠期糖尿病患者进行针对性的运动指导 |  |  |  |  |  |
|  | 21.我能对妊娠期糖尿病患者进行正确的用药指导 |  |  |  |  |  |
|  | 22.我能教会妊娠期糖尿病患者进行血糖的自我评估与自我管理 |  |  |  |  |  |
|  | 23.我能对妊娠期糖尿病患者的突发紧急情况（如低血糖、高渗性昏迷）规范的实施急救技术 |  |  |  |  |  |
| **专业能力** | 24.在为妊娠期糖尿病的患者实施干预措施时，我能综合分析妊娠期糖尿病患者的资料，迅速有效的确定首优护理问题 |  |  |  |  |  |
|  | 25.我能结合自己的理论知识和实践经验评判性地接受相关专业人员的意见 |  |  |  |  |  |
|  | 26.我能与不同家庭背景及文化层次的妊娠期糖尿病患者及照护者进行有效沟通 |  |  |  |  |  |
|  | 27.我能及时发现工作中引起沟通不畅的问题并有针对性的提出改进措施 |  |  |  |  |  |
|  | 28.我能开展妊娠期糖尿病健康教育科普讲课 |  |  |  |  |  |
|  | 29.我能通过文献阅读、学术交流等方式主动学习妊娠期糖尿病的专业知识 |  |  |  |  |  |
|  | 30.我能开展与妊娠期糖尿病相关的课题研究或撰写相关科研论文 |  |  |  |  |  |
|  | 31.我能掌握常用的办公软件的使用方法，对妊娠期糖尿病患者的资料进行收集和整理 |  |  |  |  |  |
| **个人态度** | 32.我能主动为妊娠期糖尿病患者及家属提供热情周到的护理服务 |  |  |  |  |  |
|  | 33.我能以积极的方式处理妊娠期糖尿病患者管理工作中遇到的问题 |  |  |  |  |  |
|  | 34.我具备慎独精神，能时刻规范地进行各项护理工作 |  |  |  |  |  |
|  | 35.我能以“南丁格尔”精神来指引自己在护理岗位上实现个人价值 |  |  |  |  |  |

**Questionnaire for evaluating management of GDM patients among obstetric nurses**

Dear nursing colleagues,

This is a survey questionnaire regarding the management capabilities of obstetric nurses for gestational diabetes mellitus (GDM) patients. The main purpose is to understand the current state of care provided by obstetric nurses for GDM patients. Your participation is completely voluntary, you have the right to refuse to participate or withdraw from the survey at any time without any penalty or negative consequences. If you agree to participate, please respond according to your own situation! The information you provide will only be used for academic statistical analysis and will not reveal your personal identity in any way. We guarantee the confidentiality of all personal information of participants in the study, so please feel free to participate!

Thank you very much for your cooperation, I wish you a happy life!

College of Nursing, Henan University of Science and Technology

Yongyan Zhu

**Part I: General information of obstetric nurses**

1. Gender: ○Female ○Male

2. Age:

3. Ethnic Group: ○Han ○Minority

4. Marital status: ○Married ○Unmarried ○Divorced or other

5. Birth history: ○Yes ○No

6. Highest education: ○Secondary school ○Junior College ○Undergraduate

○Master degree or above

7. Job title: ○Nurse ○Senior nurse ○Supervisor ○Deputy Chief nurse and above

8. Years of work in obstetric care:

9. Work experience in endocrinology: ○Yes ○No

10. The number of training related to gestational diabetes:

○No ○1/year ○2-3/year ○>3 / year

(Training criteria: the trainer is a doctor, dietitian or nurse supervisor above the nursing staff, each training time is not less than 30 minutes)

**Part II:《Obstetric Nurses' Ability to Manage GDM patients》**

Please mark "√" in the box for each item according to your actual situation.

| [Dimensionality](javascript:;) | Item | Ability classification | | | | |
| --- | --- | --- | --- | --- | --- | --- |
|  |  | Completely incapable | Poor capable | Average capable | Strong capable | Highly capable |
| Professional Knowledge Dimension  (KD) | 1. I am familiar with the risk factors of GDM and its adverse effects on both the mother and the child. |  |  |  |  |  |
|  | 2. I know the diagnostic criteria and glycemic control goals for GDM very well. |  |  |  |  |  |
|  | 3. I am proficient in the exercise guidance knowledge of GDM. |  |  |  |  |  |
|  | 4. I am proficient in dietary guidance for GDM. |  |  |  |  |  |
|  | 5. I am familiar with the medication knowledge and adverse reactions of GDM. |  |  |  |  |  |
|  | 6. I am aware of the common mother and child complications of GDM (such as hypoglycemia, ketoacidosis, hypertonic coma, macrosomia, etc.) |  |  |  |  |  |
|  | 7. I am aware of the normal range and clinical significance of blood glucose and glycosylated hemoglobin in GDM patients. |  |  |  |  |  |
|  | 8. I am aware of the timing, normal range and clinical significance of blood glucose monitoring in newborns born with GDM. |  |  |  |  |  |
|  | 9. I am aware of the clinical manifestations and nursing measures of neonatal hypoglycemia in GDM patients. |  |  |  |  |  |
|  | 10. I am aware of the content of postpartum health education and review guidance for GDM. |  |  |  |  |  |
|  | 11. I possess the psychological knowledge required to assess the psychological characteristics of patients with GDM. |  |  |  |  |  |
|  | 12. I am knowledgeable about relevant healthcare policies, regulations, and ethical standards. |  |  |  |  |  |
| Professional Skill Dimension (SD) | 13. I can do health risk assessments for GDM patients. |  |  |  |  |  |
|  | 14. I am capable of providing preventive health guidance for patients with high-risk factors for GDM. |  |  |  |  |  |
|  | 15. I can accurately assess the lifestyle of GDM patients. |  |  |  |  |  |
|  | 16. I am proficient in operating techniques (such as blood sugar monitoring, insulin injection, etc.) for GDM patients. |  |  |  |  |  |
|  | 17. I can master the monitoring skills of fetal heart and fetal movement in GDM patients. |  |  |  |  |  |
|  | 18. I can use psychological knowledge and skills to provide effective psychological intervention for GDM patients. |  |  |  |  |  |
|  | 19. I can provide targeted dietary guidance for GDM patients. |  |  |  |  |  |
|  | 20. I can provide targeted exercise guidance to GDM patients. |  |  |  |  |  |
|  | 21. I can give proper medication guidance to GDM patients. |  |  |  |  |  |
|  | 22. I can teach GDM patients how to self-assess and manage their blood glucose levels. |  |  |  |  |  |
|  | 23. I am able to implement emergency techniques for GDM patients in emergency situations (such as hypoglycemia, hypertonic coma). |  |  |  |  |  |
| Professional Ability (AbD) | 24. When implementing interventions for GDM patients, I can comprehensively analyze the data of GDM patients and quickly and effectively identify the optimal care issues. |  |  |  |  |  |
|  | 25. I can combine my theoretical knowledge and practical experience to accept the opinions of relevant professionals critically. |  |  |  |  |  |
|  | 26. I am able to communicate effectively with GDM patients and their caregivers from different family backgrounds and cultural levels. |  |  |  |  |  |
|  | 27. I was able to find the problems that caused the miscommunication at work in time and put forward the improvement measures accordingly. |  |  |  |  |  |
|  | 28. I can give lectures on GDM health education. |  |  |  |  |  |
|  | 29. I can actively learn professional knowledge about GDM through literature reading and academic exchanges. |  |  |  |  |  |
|  | 30. I can conduct research or write research papers related to GDM. |  |  |  |  |  |
|  | 31. I can master the use of commonly used office software and collect and sort out the data of GDM patients. |  |  |  |  |  |
| Personal Attitude (AtD) | 32. I can take the initiative to provide warm and considerate care to GDM patients and their families. |  |  |  |  |  |
|  | 33. I am able to deal with the problems encountered in the management of GDM patients in a positive way. |  |  |  |  |  |
|  | 34. I have the spirit of being prudent and independent, and can carry out all kinds of nursing work regularly. |  |  |  |  |  |
|  | 1. I can use the spirit of "Nightingale" to guide myself to realize personal value in nursing post. |  |  |  |  |  |
